# Role of CXCR1 and Interleukin-8 in Methamphetamine-Induced Neuronal Apoptosis

**DOI:** 10.3389/fncel.2018.00230

**Published:** 2018-08-03

**Authors:** Si-Hao Du, Wei Zhang, Xia Yue, Xiao-Qing Luo, Xiao-Hui Tan, Chao Liu, Dong-Fang Qiao, Huijun Wang

**Affiliations:** ^1^School of Forensic Medicine, Southern Medical University, Guangzhou, China; ^2^Nanfang Hospital, Southern Medical University, Guangzhou, China; ^3^Guangzhou Forensic Science Institute, Guangzhou Public Security Bureau, Guangzhou, China

**Keywords:** methamphetamine, CXCR1, IL-8, neurotoxicity, apoptosis, astrocytes

## Abstract

Methamphetamine (METH), an extremely and widely abused illicit drug, can cause serious nervous system damage and social problems. Previous research has shown that METH use causes dopaminergic neuron apoptosis and astrocyte-related neuroinflammation. However, the relationship of astrocytes and neurons in METH-induced neurotoxicity remains unclear. We hypothesized that chemokine interleukin (IL) eight released by astrocytes and C-X-C motif chemokine receptor 1 (CXCR1) in neurons are involved in METH-induced neuronal apoptosis. We tested our hypothesis by examining the changes of CXCR1 in SH-SY5Y cells and in the brain of C57BL/6 mice exposed to METH by western blotting and immunolabeling. We also determined the effects of knocking down CXCR1 expression with small interfering ribonucleic acid (siRNA) on METH-exposed SH-SY5Y cells. Furthermore, we detected the expression levels of IL-8 and the nuclear factor-kappa B (NF-κB) pathway in U87MG cells and then co-cultured the two cell types to determine the role of CXCR1 and IL-8 in neuronal apoptosis. Our results indicated that METH exposure increased CXCR1 expression both *in vitro* and *in vivo*, with the effects obtained *in vitro* being dose-dependent. Silencing of CXCR1 expression with siRNAs reduced the expression of cleaved caspase-3, cleaved poly (ADP-ribose) polymerase (PARP), and other related proteins. In addition, IL-8 expression and release were increased in METH-exposed U87MG cells, which is regulated by NF-κB pathway. Neuronal apoptosis was attenuated by siCXCR1 after METH treatment in the co-cultured cells, which can be reversed after exposure to recombinant IL-8. These results demonstrate that CXCR1 plays an important role in neuronal apoptosis induced by METH and may be a potential target for METH-induced neurotoxicity therapy.

**Highlights**

– Methamphetamine exposure upregulated the expression of CXCR1.
– Methamphetamine exposure increased the expression of interleukin-8 through nuclear factor-kappa B pathway.
– Activation of CXCR1 by interleukin-8 induces an increase in methamphetamine-related neuronal apoptosis.

## Background

Methamphetamine (METH) is a highly addictive psychoactive drug that can have a significant injurious effect on the central nervous system (CNS). The abuse of METH has become more common than that of either heroin or cocaine and has thus placed great pressure on the economy and social order (Dinis-Oliveira et al., 2012).

Clinical and animal model studies of METH have shown that long-term consumption of METH results in significant damage to the dopaminergic system, including direct pharmacological damage of the sensitive brain region (Kita et al., 2009; Zhang et al., 2013), oxidative stress and inflammatory lesions, along with the destruction of dopaminergic neurons (Huang et al., 2015).

Our previous studies and other laboratories’ research found that METH can cause neuronal apoptosis through P53 upregulated modulator of apoptosis (PUMA), insulin-like growth factor-binding protein 5 (IGFBP5), and other pathways (Qiao et al., 2014; Carmena et al., 2015; Chen C. et al., 2016; Mendieta et al., 2016). However, the treatment of primary astrocytes with METH showed that the nuclear import of nuclear factor-kappa B (NF-κB) could be increased through Toll-like receptor 4, leading increased release of pro-inflammatory cytokines, such as interleukin (IL)-1β and IL-18 (Du et al., 2017). Our recent study showed that the regulation of chemokines through a NF-κB/IL-8/C-X-C motif chemokine receptor 1 (CXCR1) pathway may play an important role in METH-induced neuronal apoptosis.

The pro-inflammatory IL-8 is a multi-functional CXC chemokine of about 75 amino acids in length (Waugh and Wilson, 2008; Citro et al., 2015). Numerous stresses, including inflammatory signals such as IL-1β and tumor necrosis factor alpha, environmental stresses (including hypoxia and/or reactive oxygen species) or surgical damage can induce IL-8 expression (Hoffmann et al., 2002; Sordillo and Helson, 2015). It turns out that both astrocytes and neurons produce IL-8 *in vitro*, stimulated by pro-inflammatory cytokines (Vlahopoulos et al., 1999; Waugh and Wilson, 2008; Kou et al., 2011). It has also been demonstrated that exposure of the cells to CpG oligodeoxynucleotides and lipopolysaccharides (LPS) increased the expression of IL-8 through mitogen-activated protein kinase (MAPK)-dependent and NF-κB-independent pathways (Kim et al., 2005). Two high-affinity receptors for IL-8 in humans are designated CXCR1 (also called IL-8R-A) and CXCR2 (Lee et al., 1992; Loetscher et al., 1994). The two receptors are expressed not only on leukocytes and tumor cells but also on neutrophils, monocytes, macrophages, basophils, natural killer T-cells and others normal cells; in addition, a lower percentage of T-cells express CXCR1/2 (Flynn et al., 2003; Brat et al., 2005). Additionally, it has been indicated that cells in CNS express chemokine receptors, including CXCR1 and CXCR2 (Dorf et al., 2000).

Chemokine IL-8, also known as CXCL8, exerts its biological effects by binding to the specific G protein-coupled receptors of CXCR1 and CXCR2 (Citro et al., 2015). After their internalization (i.e., via MAPK, phosphoinositide 3-kinase (PI3K), protein kinase c and Src tyrosine kinase, etc.), a wide range of intracellular pathways are activated (Takahashi et al., 2007; Citro et al., 2015). Therefore, in certain pathological conditions, CXCR1 is crucial in inflammatory injury and may be a promising target for neuronal apoptosis (Clunes and Boucher, 2007; Eltzschig and Eckle, 2011). However, the role of CXCR1 in neuronal apoptosis induced by METH remains to be investigated.

We hypothesized that CXCR1 would mediate METH-induced neuronal apoptosis and that the blockade of CXCR1 expression might partially protect against METH-induced neuronal apoptosis. In this study, we examined CXCR1 levels, and they showed elevated expression in METH poisoning models *in vivo* and *in vitro*. We also found that METH treatment of astrocytes resulted in increased expression of IL-8 through the activation of the NF-κB pathway. The apoptosis of neurons increased with the addition of exogenous IL-8, while the apoptosis of neurons was reduced after the silencing of CXCR1. These findings indicated that CXCR1 activation is important in the process of METH-induced neuronal apoptosis. Our study showed that CXCR1 may be a potential target in the mitigation of neuronal damage and apoptosis induced by METH.

## Materials and Methods

### Materials

High Glucose Dulbecco’s modified Eagle’s medium (DMEM), fetal bovine serum (FBS), opti-MEM, Lipofectamine 3000 and trypsin were purchased from Gibco (Carlsbad, CA, USA). METH (>99% purity) was obtained from the National Institutes for the Control of Pharmaceutical and Biological Products (Beijing, China). Anti-cleaved poly (ADP-ribose) polymerase (PARP), anti-cleaved caspase3, anti-IKK-α, anti-IKK-β, anti-phospho-IKKα/β, anti-phospho-NF-κB, anti-IκB and anti-rabbit and mouse IgG (H + L), F(ab’)2 fragment (Alexa Fluor 555 conjugated) were purchased from the Cell Signaling Technology (Boston, MA, USA). Anti-NeuN was purchased from Abcam (Cambridge, UK). Anti-NF-κB, anti-Bax, anti-Bcl-2 and anti-IL-8 were purchased from ABclonal Inc (College Park, MD, USA). Anti-β-actin and goat anti-mouse and rabbit IgG (H + L)-HRP were purchased from Beijing Ray Antibody Biotech (Beijing, China). Anti-CXCR1 was purchased from Bioss (Beijing, China). Fluorescein (FITC)-conjugated goat anti-mouse and rabbit IgG were purchased from DingGuo (Beijing, China). siRNAs for CXCR1 was purchased from the Shanghai GenePharma Company Limited (Shanghai, China). Human IL-8 ELISA kit was purchased from Cusabio Biotech (Wuhan, China). Super ECL Assay was purchased from KeyGEN Biotech (Nanjing, China). Recombinant IL-8 was purchased from PerproTech (Rocky Hill, NJ, USA). Bay 11-7082 ((E)3-[(4-methylphenyl)-sulfonyl]-2-propenenitrile; a NF-κB inhibitor) was purchased from SelleckChem (Houston, TX, USA). Other reagents, unless specifically mentioned, were purchased from Sigma-Aldrich (St. Louis, MO, USA).

### Animal Protocol

Healthy adult male C57BL/6 mice (18–22 g, 6–8 weeks old) were purchased from Southern Medical University Experimental Animal Center (Guangzhou, China) and placed individually in a temperature-controlled tub cage (approximately 22°C) with 12 h of light/dark cycle room. All procedures involving animals were performed in accordance with the ethical standards of Ethics Committee of Nanfang Hospital, Southern Medical University and with the 1964 Helsinki Declaration and its later amendments or comparable ethical standards. This article does not contain any studies with humans performed by any of the authors. Mice were placed in animal facilities for 1 week before use to make them accustomed. Mice were randomly divided into two groups (*n* = 3/group): saline control group and METH subacute exposure group. METH is dissolved in physiological saline. Mice in the subacute exposure group received an intraperitoneal (i.p.) METH injection (15 mg/kg/injection) every 12 h for a total of eight injections. This pattern of exposure is based on our and other previous studies (Cadet et al., 2003; Krasnova and Cadet, 2009; Qiao et al., 2014; Du et al., 2017). Saline control mice were injected i.p. with a similar volume of saline. Injections were performed at the same time as the subacute exposure group. All animals survived throughout the study. Mice were euthanized 2 h after the last injection (CO_2_; followed by decapitation). Brain regions were rapidly isolated, and half hemispheres were cut and placed in 4% paraformaldehyde for 24 h for immunofluorescence experiments. The other half hemispheres were dissected to the prefrontal cortex, hippocampus, midbrain and striatum region on iced glass plates, rapidly frozen and stored at −80°C until analysis.

### Cell Culture

SH-SY5Y cells, a human neuroblastoma cell line and U87MG cells, a human primary glioblastoma cell line were purchased from the Cell Bank of Shanghai Institute for Biological Center, Chinese Academy of Science (Shanghai, China). Cells were cultured in DMEM medium containing 10% FBS and placed in a 37°C constant temperature humidification, 5% carbon dioxide cell culture box. The culture medium was changed 1 or 2 days. Cells were passaged to 6-well plate when they reached about 80% to 90%.

### Methamphetamine and Inhibitor Treatment

Once cells were reached about 80%, medium was changed to non-serum medium. Then cells were exposed to 0, 0.5, 1.0, 1.5, 2.0 and 2.5 mM METH in U87MG cells or SH-SY5Y cells for 24 h. This concentration range was selected based on our and others previous studies (Cisneros and Ghorpade, 2014; Zhang et al., 2015; Cao et al., 2016) and this concentration covers the non-toxic, sub-toxic and 50% lethal concentrations of METH (Chen C. et al., 2016). In the experiments with inhibitor Bay 11-7082, the cells were pre-cultured for 12 h with 10 μM Bay 11–7082 and then incubated with 2.0 mM METH for 24 h. The concentration of Bay 11–7082 was selected based on earlier studies (Pierce et al., 1997; Zanotto-Filho et al., 2011) and this concentration had optimal inhibition effects in our experiment.

### Co-culture of U87MG Cells and SH-SY5Y Cells

U87MG cells and SH-SY5Y cells were cultured to a density of 90% and passaged into transwell dishes (0.4 μM; purchased from Corning (Corning, NY, USA)). SH-SY5Y cells were plated in the upper chamber of the transwell plate and U87MG cells were spread on the bottom of the transwell plate and cultured to a density of 70% to 80% respectively. The upper chamber was then placed above the lower chamber and exposed to 2.0 mM of METH for 24 h.

### Western Blot Analysis

We used a Radio Immunoprecipitation Assay (RIPA) cleavage method to extract cell protein. Protein concentration was determined by BCA-100 protein quantification kit, which was purchased from Biosharp (Hefei, China). A sample of 50 μg of protein were taken and separated by 10% to 15% polyacrylamide gel electrophoresis. The protein was transferred to polyvinylidenefluoride (PVDF) membrane (Millipore, Billerica, MA, USA) and then the PVDF membranes were incubated for 1 h at room temperature (RT) in 5% (w/v) skim milk in tris-buffered saline containing 0.1% Tween 20 (TBST), followed by incubated overnight with a primary antibody at 4°C (1:500–1:1,000) in TBST. Next, the PVDF membranes were washed for three times with TBST and then incubated for 1 h at RT with secondary antibody (1:10,000) followed by three times washing with TBST. The membranes were developed with 200 μl of super ECL assay. The band intensities were quantitated by Gel-Pro analyzer (Media Cybernetics Inc., Rockville, MD, USA). We used β-actin as our reference index, and each experiment was repeated three times, with the most representative results presented in the current manuscript.

### Small Interfering Ribonucleic Acid and Transient Cell Transfection

Transfection was performed as described previously (Du et al., 2017). The sequences of small interfering ribonucleic acid (siRNA) are shown in Table 1. Twenty-four hours before transfection, 4−5 × 10^5^ SH-SY5Y cells were seeded on 6-well culture plates, and 2 ml of DMEM medium containing 10% FBS was added to each well. Then, 20 μMol of CXCRl siRNA or control siRNA (siNC) (GenePharma, Shanghai, China) and 5 μl of lipofectamine 3000 reagent were added to 1 ml opti-MEM medium. The mixed solution was incubated at RT for 20 min, and then added to each well of the culture plate in conjunction with gentle shaking and mixing. The cell culture plates were placed in incubator for 6–12 h and then 1 ml complete medium was added in each well.

**Table 1:** The sequences of small interfering ribonucleic acids (siRNAs) used in the present study.

Gene	Number	The sequence of siRNA (5′-3′)
CXCR1	1	CCGCCAGGCUUACCAUCCAAACAAU
CXCR1	2	UCGUGCCGCUGUUUGUCAUGCUGUU
Negative control	1	UUCUCCGAACGUGUCACGUTT

### Double Immunofluorescence Labeling

Double immunofluorescence labeling was performed as described previously (Du et al., 2017). To determine CXCR1 expression levels in SH-SY5Y cells and mice midbrain samples, double immunofluorescence labeling on cells and frozen sections of adult mice midbrains was performed. For immunolabeling, SH-SY5Y cells were seeded on glass bottom cell culture dishes (NEST, Shanghai, China). Cells were fixated in 4% paraformaldehyde. Then cells were incubated with blocking buffer (10% BSA and 0.05% Triton X-100) for 30 min at RT, with the primary antibody (anti-NeuN dilution of 1:200 and anti-CXCR1 dilution of 1:100) overnight at 4°C, and then with the secondary antibody for 1 h at RT (FITC conjugated anti-mouse or rabbit IgG dilution of 1:50, Alexa Fluor 555 conjugated anti-mouse or rabbit IgG dilution of 1:200). All incubation solutions were prepared using PBS supplemented with 10% BSA and 0.05% Triton X-100. 4’,6-diamidino-2-phenylindole (DAPI) was used to stain nucleus. The frozen tissue sections were incubated with cold acetone for 15 min to remove the embedding agent. The rest of the steps are the same as for the cells. Microphotographs were taken using fluorescence microscopy (A1+/A1R+; Nikon). All digital images were processed using the same settings to improve the contrast.

### Enzyme Linked Immunosorbent Assay (ELISA)

ELISA was performed as Human IL-8 ELISA kit directions described (Cusabio Biotech, China). Briefly, add 100 μl of standard or test sample per well and incubate for 2 h at 37°C. Remove the liquid of each well and add 100 μl of primary antibody and incubate for 1 h at 37°C. Remove the liquid, wash the plate three times with PBS for 2 min each time, add 100 μl horseradish peroxidase labeled avidin secondary antibody, and incubate for 1 h at 37°C. Remove the liquid, wash the plate three times with PBS, add 90 μl of the substrate solution. Incubate for 15–30 min at 37°C to avoid light. Add 50 μl of stop solution to stop the reaction. Determine the optical density of each well within 5 min, using a microplate reader set to 450 nm.

### Terminal Deoxynucleotidyl Transferase dUTP Nick End-Labeling (TUNEL) Staining

The DNA fragments of the apoptotic neurons were detected according to the instructions of a terminal deoxynucleotidyl transferase dUTP nick end labeling (TUNEL) fluorescent reagent. First, cells were fixed with 4% paraformaldehyde for 10 min at RT and then incubated for 1 h in cell culture incubator by adding terminal deoxynucleotidyl transferase enzyme conjugated to fluorescein, which was followed by the introduction of DAPI for nuclear count staining. The cross-sectional fragment was observed under a fluorescence microscope. Treatment in the control group was consistent with the addition of terminal deoxynucleotide transferase. TUNEL-positive cells and DAPI-positive cells were counted.

### Statistical Analysis

Statistical analysis of data was performed with Student’s *t*-test and one-way analysis of variance (ANOVA) followed by Least Significant Difference (LSD) *post hoc* analyses using SPSS 20.0 software (IBM Corporation, Armonk, NY, USA). Data given in the text are expressed as mean ± standard deviation (SD). The value of *P* < 0.05 was considered statistically significant.

## Results

### Methamphetamine-Induced CXCR1 Expression in Neurons

**Figure 1:**
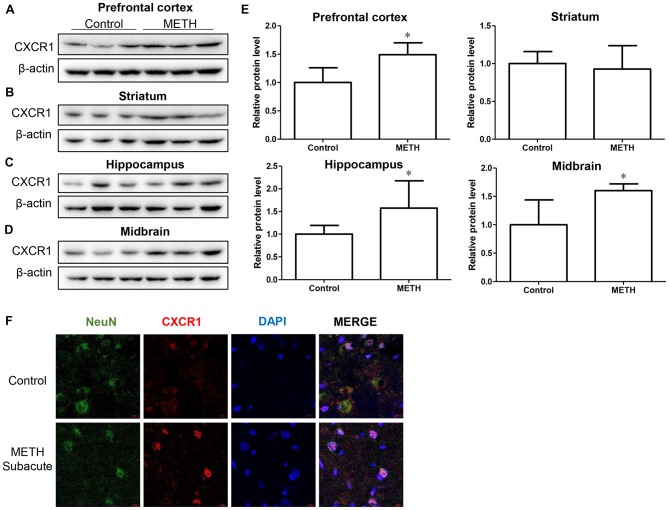
Methamphetamine (METH) increased C-X-C motif chemokine receptor 1 (CXCR1) expression in the brain of male C57BL/6 mice. Male C57BL/6 mice were divided randomly into two groups: experimental group and control group (*n* = 3/group). Animals were injected intraperitoneally (i.p.) with METH (eight injections, 15 mg/kg/injection, at 12-h intervals) or saline. The prefrontal cortex, hippocampus, midbrain and striatum tissues were separated on ice at 2 h after the last dosing. Western blot **(A–D)** and quantitative analyses **(E)** were performed to determine CXCR1 expression. β-actin was used as a loading control. Fold induction relative to the control group is shown. *Represents a significant difference as compared with the control or vehicle-treated group, *p < 0.05. Results are expressed as the mean ± standard deviation (SD) of three experiments. Data were analyzed with Student’s *t*-test. Immunolabeling and confocal imaging analyses **(F)** showed increased CXCR1 expression in the midbrain of METH-exposed mice in comparison with in the controls. Scale bar, 15 μM.

In order to determine the role of CXCR1 in METH-triggered neurotoxicity, a mouse model treated with METH (eight injections, 15 mg/kg/injection, given at 12-h intervals) was used to detect the expression of CXCR1 *in vivo*. We separated the brain regions of prefrontal cortex, hippocampus, midbrain and striatum for further testing. Western blot results showed that CXCR1 protein levels in the prefrontal cortex, hippocampus and midbrain were higher in the subacute exposure group than in the control group (Figures 1A,C–E), while the expression of CXCR1 in the striatum showed no obvious effect between the two groups (Figures 1B,E). Immunofluorescence staining results also showed that the expression of CXCR1 was increased in the midbrain of METH-treated C57BL/6 mice (Figure 1F). In addition, we also observed co-localization of CXCR1 with neurons, indicating that CXCR1 is mainly expressed in the neurons of the midbrain.

**Figure 2:**
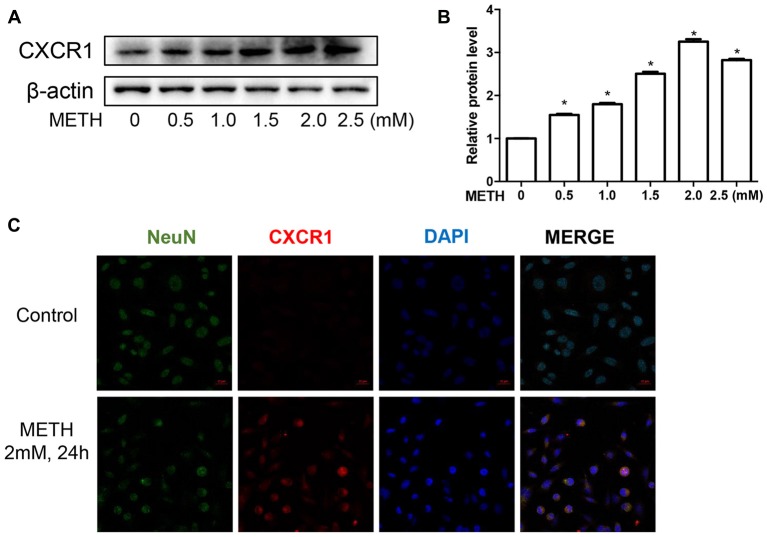
METH increased CXCR1 expression in neurons. SH-SY5Y cells were exposed to 0.5 mM, 1 mM, 1.5 mM, 2 mM and 2.5 mM of METH for 24 h **(A)** or 2 mM of METH for 24 h **(C)**. Western blot **(A)** and quantitative analyses **(B)** were performed to determine CXCR1 protein expression. β-actin was used as a loading control. Fold induction relative to the control group is shown. *Represents a significant difference as compared with the control or vehicle-treated group, *p < 0.05. Results are expressed as the mean ± SD of three experiments. Data were analyzed with one-way ANOVA followed by least significant difference (LSD) *post hoc* analyses. Immunolabeling and confocal imaging analyses **(C)** showed elevated CXCR1 expression in the SH-SY5Y cells treated with METH in comparison with in the controls. Scale bar, 50 μM.

To validate this phenomenon *in vitro*, we selected SH-SY5Y cells to determine the expression of CXCR1. SH-SY5Y cells were treated with different concentrations of METH—specifically, 0 mM, 0.5 mM, 1 mM, 1.5 mM, 2 mM, or 2.5 mM—and western blot analysis was performed to detect CXCR1 expression. The results showed that CXCR1 protein expression was increased in a dose-dependent manner in the SH-SY5Y cells (Figures 2A,B). CXCR1 protein expression was 3.25-fold higher in the 2.0 mM METH-treated SH-SY5Y cells for 24 h than in the control cells. Immunofluorescence staining results demonstrated the same results (Figure 2C). These findings suggest that METH exposure induces CXCR1 protein expression both *in vivo* and *in vitro*.

### Importance of CXCR1 Induction in Neuronal Apoptosis Induced by Methamphetamine

**Figure 3:**
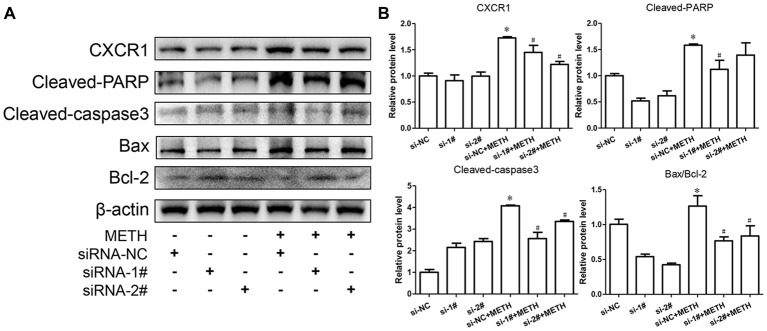
Silencing of CXCR1 expression with small interfering ribonucleic acid (siRNA)-attenuated METH-induced neuronal apoptosis. SH-SY5Y cells were transfected with siRNA targeting CXCR1 or control siRNA for 24 h, followed by 2 mM of METH treatment for 24 h. Western blot **(A)** and quantitative analyses **(B)** were performed to determine CXCR1, cleaved caspase-3, cleaved PARP, Bax and Bcl-2 protein expression. β-actin was used as a loading control. Fold induction relative to the control group is shown. *Represents a significant difference as compared with the non-METH-treated group, *p < 0.05. ^#^Represents a significant difference as compared with the scrambled + METH treated group, ^#^*p* < 0.05. Results are expressed as the mean ± SD of three experiments. Data were analyzed with one-way ANOVA followed by LSD *post hoc* analyses.

To confirm the relationship of CXCR1 and neurotoxicity induced by METH, we designed two siRNA sequences for SH-SY5Y cells to silence CXCR1 expression. The results showed that both of the siRNA sequences could attenuate CXCR1 induction effectively in SH-SY5Y cells (Figures 3A,B). After pre-treatment with siRNA#1 or siRNA#2 of CXCR1, the cells were treated with METH (2 mM, 24 h). CXCR1 protein expression decreased respectively, as compared with in the siRNA-NC + METH treatment group (Figures 3A,B).

To evaluate the protective effect of CXCR1 silencing from METH-induced neuronal apoptosis, we determined the expression of cleaved caspase-3 and cleaved PARP by western blot analyses. We found that METH increased the level of cleaved caspase-3 and cleaved PARP obviously; this effect was significantly inhibited after CXCR1 silencing (Figures 3A,B). We then sought to examine whether Bcl-2/Bax are involved in METH-induced CXCR1-mediated neuronal apoptosis. Western blot analyses were performed to detect changes in Bcl-2 and Bax expression levels with and without CXCR1 knockdown. The results showed that the expression level of Bcl-2, an antiapoptotic factor, was increased following CXCR1 knockdown in comparison with in the si-NC + METH treatment group, while the expression level of Bax, a proapoptotic factor, was decreased (Figures 3A,B). Taken together, these results suggest that the silencing of CXCR1 inhibits the Bcl-2/Bax expression and that CXCR1 participates in the mitochondria-mediated apoptosis pathway triggered by METH.

### Methamphetamine Increases the Expression of Chemokine IL-8 via Nuclear Translocation of NF-κB in Astrocytes

**Figure 4:**
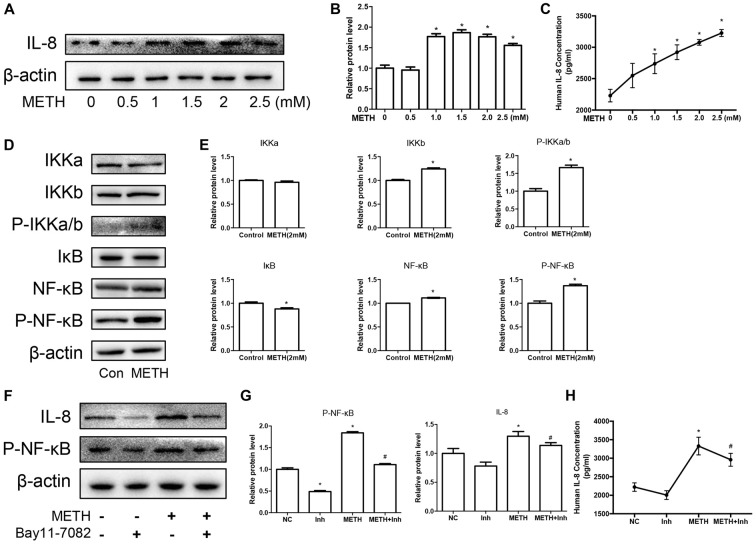
METH increases the expression of chemokine interleukin (IL)-8 via nuclear translocation of NF-κB in astrocytes. U87MG cells were exposed to 0.5 mM, 1 mM, 1.5 mM, 2 mM and 2.5 mM of METH for 24 h **(A)** or 2 mM of METH for 24 h **(D)**. **(F)** U87MG cells were exposed to Bay 11–7082 (10 μM) for 12 h prior to METH (2 mM) treatment as indicated. Western blot **(A,D,F)** and quantitative analyses **(B,E,G)** were performed to determine IL-8, IKK-α, IKK-β, phospho-IKK-α/β, IκB, NF-κB and phospho-NF-κB protein expression. β-actin was used as a loading control. ELISA **(C,H)** was performed to detect the concentration of IL-8 in culture medium supernatant. Fold induction relative to the control group is shown. *Represents a significant difference as compared with the non-METH-treated group, *p < 0.05. ^#^Represents a significant difference as compared with the scrambled + METH treated group, ^#^*p* < 0.05. Results are expressed as the mean ± SD of three experiments. Data in **(A,C,F,G)** were analyzed with one-way ANOVA followed by LSD *post hoc* analyses; data in **(D)** were analyzed with Student’s *t*-test.

We have demonstrated that the expression of CXCR1 is upregulated in METH-treated SH-SY5Y cells and may be involved in METH-induced apoptosis. IL-8 is a cytokine secreted mostly by macrophages and epithelial cells. Studies have demonstrated that astrocytes can also secrete IL-8, so we applied METH in human-derived U87MG cells and found that the expression of IL-8 was increased in a dose-dependent manner (Figures 4A–C). Specifically, at 2.0 mM, IL-8 protein expression in the U87MG cells was ~1.77-fold higher than in the control cells. Similar effects were observed in the medium of the cells. The concentration of IL-8 in the medium was ~1.38-fold higher than in the control (Figure 4C). According to our previous study, the expression of transcription factor NF-κB was upregulated in the METH-treated primary cultured C57BL/6 mouse astrocytes. To verify whether the NF-κB pathway is activated in U87MG cells, cells were exposed to 2.0 mM of METH and then western blot analysis was performed to detect the expressions of NF-κB and phosphor-NF-κB. The results showed that NF-κB protein expression was increased slightly and that phosphor-NF-κB protein expression was significantly increased (Figures 4D,E) in the METH-treated U87MG cells as compared with in the control. The expression of IκB kinase (IKK)-α did not change significantly, but the expressions of both IKK-β and phosphor-IKK-α/β were increased, respectively (Figures 4D,E). IκB was significantly decreased in the METH-treated U87MG cells. Taken together, these results showed that METH exposure induces the activation of the NF-κB pathway in U87MG cells. To confirm whether the release and expression of IL-8 is associated with NF-κB pathway activation in METH-treated U87MG cells, the inhibitor of NF-κB, Bay 11-7082, was administered to U87MG cells and western blot was performed. Results showed that a blockade of NF-κB reduces METH-induced IL-8 expression in U87MG cells (Figures 4F,G). We also detected the concentration of IL-8 in culture medium by ELISA; similar effects were observed (Figure 4H).

### Co-culture of U87MG Cells and SH-SY5Y Cells Reduced the Apoptosis of SH-SY5Y Cells Induced by Methamphetamine

**Figure 5:**
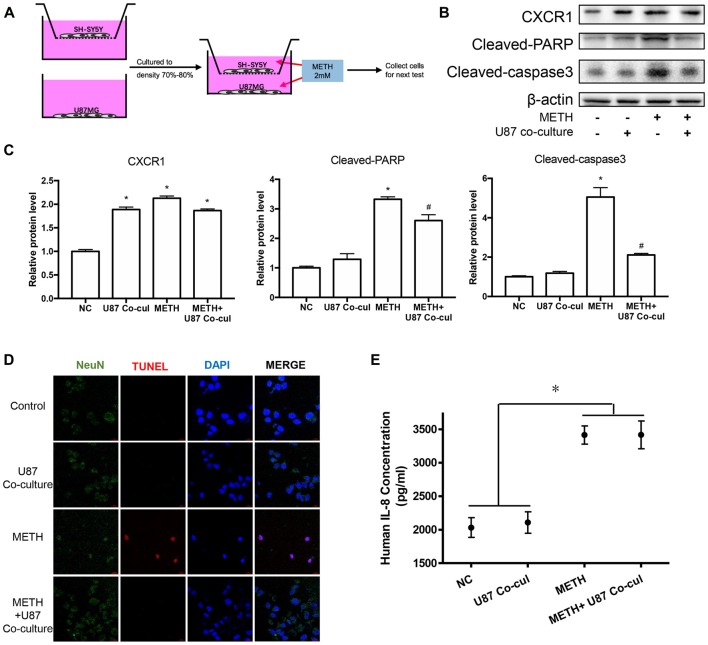
Co-culture of U87MG cells and SH-SY5Y cells reduced the apoptosis of SH-SY5Y cells induced by METH. **(A)** Co-culture pattern diagram. U87MG cells and SH-SY5Y cells were cultured to a density of 90% and passaged into Transwell dishes. SH-SY5Y cells were plated in the upper chamber of the Transwell plate and U87MG cells were spread on the bottom of the Transwell plate and cultured to a density of 70% to 80% respectively. The upper chamber was then placed above the lower chamber and exposed to 2.0 mM of METH for 24 h. Western blot **(B)** and quantitative analyses **(C)** were performed to determine CXCR1, cleaved caspase-3 and cleaved PARP protein expression. β-actin was used as a loading control. Fold induction relative to the control group is shown. *Represents a significant difference as compared with the negative control group, *p < 0.05. ^#^Represents a significant difference as compared with the scrambled + METH treated group, ^#^*p* < 0.05. Results are expressed as the mean ± SD of three experiments. Data were analyzed with one-way ANOVA followed by LSD *post hoc* analyses. terminal deoxynucleotidyl transferase dUTP nick end-labeling (TUNEL) staining and confocal imaging analysis **(D)** was performed to evaluate the SH-SY5Y cells apoptosis. Scale bar, 15 μM. ELISA **(E)** was performed to detect the concentration of IL-8 in culture medium supernatant.

To explore the effects of METH on neurons in a co-culture mode of astrocytes and neurons, we constructed a cell co-culture model using transwell chambers (Figure 5A). Western blot analysis was performed to detect the expression of neuronal apoptosis-related protein cleaved caspase-3 and cleaved PARP. Our results revealed that cleaved caspase-3 and cleaved PARP expression was significantly attenuated in the co-cultured cells after METH exposure (Figures 5B,C). Apoptosis cells were also observed with TUNEL staining (Figure 5D). However, the expression of CXCR1 showed no significant difference between in the untreated co-culture group and in the METH-treated co-culture group, which indicated that CXCR1 protein expression is induced by another way in the co-culture model of U87MG cells and SH-SY5Y cells. We also detected the concentration of IL-8 in culture medium by ELISA (Figure 5E). Results showed that IL-8 expressions were increased in METH-exposed group, whether co-culture with SH-SY5Y or not.

### Silencing of CXCR1 Expression With siRNA Reduced Methamphetamine-Induced Caspase-3 and PARP Activation in Co-cultured Cell Models

**Figure 6:**
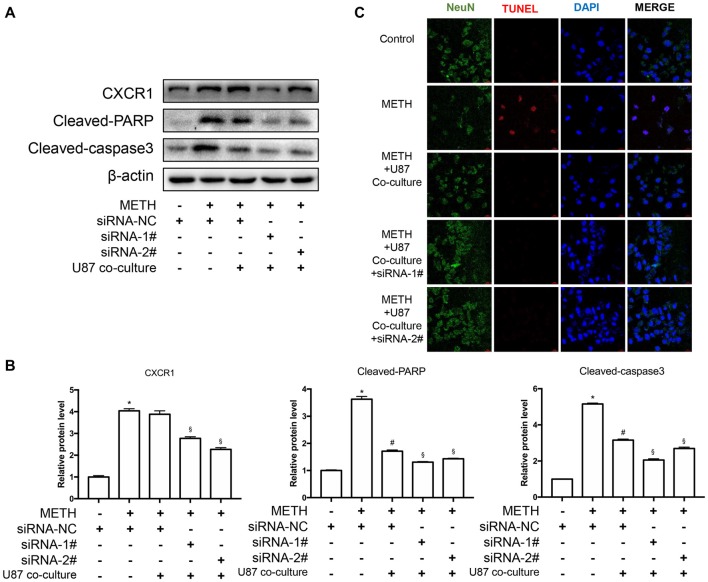
Silencing of CXCR1 expression with siRNA-reduced METH-induced caspase-3 and PARP activation in co-cultured cell models. **(A)** U87MG cells and SH-SY5Y cells were co-cultured in the same manner as before. Before METH treatment, SH-SY5Y cells were transfected with siRNA targeting CXCR1 or control siRNA for 24 h followed by co-cultured with U87MG cells. Simultaneously, 2 mM of METH was added in the medium for 24 h. Western blot **(A)** and quantitative analyses **(B)** were performed to determine CXCR1, cleaved caspase-3 and cleaved PARP protein expression. β-actin was used as a loading control. Fold induction relative to the control group is shown. *Represents a significant difference as compared with the negative control group, *p < 0.05. ^#^Represents a significant difference as compared with siRNA NC + METH treated group, ^#^*p* < 0.05. ^§^Represents a significant difference as compared with siRNA NC + METH treated +U87MG cells co-culture group, ^§^*p* < 0.05. Results are expressed as the mean ± SD of three experiments. Data were analyzed with one-way ANOVA followed by LSD *post hoc* analyses. TUNEL staining and confocal imaging analysis **(C)** was performed to evaluate the SH-SY5Y cells apoptosis. Scale bar, 15 μM.

To assess the apoptosis levels of co-culture cell models treated by METH with or without CXCR1 siRNA transfection, western blot analysis was conducted. The results are shown in Figures 6A,B. The expression of CXCR1 in SH-SY5Y cells was increased in the METH-treated group with or without U87MG cells in the co-culture as compared with in the control group, while both of the siRNA sequences could effectively knockdown the expression of CXCR1. In addition, after the treatment of siRNA of CXCR1, cleaved caspase-3 and cleaved PARP protein expression were significantly decreased in comparison with in the negative control siRNA transfection group. To confirm that silence of CXCR1 protects against METH induced SH-SY5Y cells apoptosis, TUNEL staining was performed (Figure 6C). Results suggest that blockade of CXCR1 expression reduces METH-induced apoptosis in SH-SY5Y cells.

### Interleukin-8 Increases PARP and Caspase-3 Protein Expression Induced by Methamphetamine *in Vitro*

**Figure 7:**
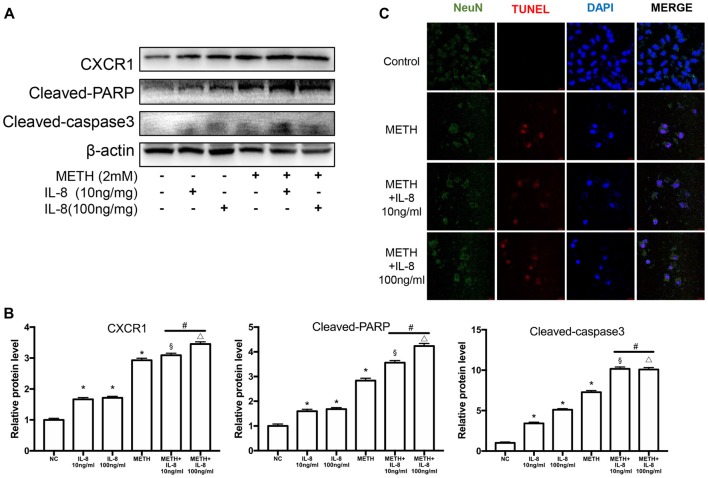
IL-8 increases PARP and caspase-3 protein expression induced by METH *in vitro*. SH-SY5Y cells were treated with 10 ng/ml or 100 ng/ml recombinant human IL-8 for 24 h, given 2 mM of METH simultaneously. Western blot **(A)** and quantitative analyses **(B)** were performed to determine CXCR1, cleaved caspase-3 and cleaved PARP protein expression. β-actin was used as a loading control. Fold induction relative to the control group is shown. *Represents a significant difference as compared with the negative control group, *p < 0.05. ^#^Represents significant difference from METH treated group without IL-8 treatment, ^#^*p* < 0.05. ^§^Represents a significant difference as compared with IL-8 (10 ng/mg) treated group, ^§^*p* < 0.05. ^△^Represents a significant difference as compared with IL-8 (100 ng/mg) treated group, ^△^*p* < 0.05. Results are expressed as the mean ± SD of three experiments. Data were analyzed with one-way ANOVA followed by LSD *post hoc* analyses. TUNEL staining and confocal imaging analysis **(C)** was performed to evaluate the SH-SY5Y cells apoptosis. Scale bar, 15 μM.

To verify the effects of IL-8 on neurons in co-culture models treated by METH, exogenous recombinant IL-8 was added to SH-SY5Y cells. We chose two concentrations (10 and 100 ng/ml) to detect the effect of IL-8 on neurons, which were obtained from previous studies (Moriceau et al., 2009; Dwyer et al., 2012; De Buck et al., 2015). In our experiments, these two concentrations of IL-8 had no apparent toxic effects on neurons alone. Then, we checked the expression levels of CXCR1, cleaved caspase-3, and cleaved PARP by use of western blot analysis. Results are shown in Figures 7A,B. The expression of CXCR1, cleaved caspase-3 and cleaved PARP were increased following recombinant IL-8 treatment. Interestingly, METH and two concentrations of recombinant IL-8 exposure together increased the expression of CXCR1, and the expression of cleaved caspase-3 and cleaved PARP was much more increased. We also investigated whether the recombinant IL-8 affects METH-induced neuronal apoptosis using TUNEL staining (Figure 7C). These results further demonstrate that IL-8-CXCR1 is involved in METH-induced neuronal apoptosis.

## Discussion

In recent years, scholars have studied the direct effects of METH from the classical dopaminergic-related neurotoxicity to more aspects. Researches showed that METH also has direct toxic effects on other cells. The results of our institute and other investigators showed that METH mediates apoptosis of the brain’s vascular endothelium through endoplasmic reticulum stress (Cai et al., 2016), mediates the opening of the blood-brain barrier (Xie et al., 2018), activation of astrocytes (Du et al., 2017), and even has some toxic effects to liver and cardiovascular system (Chen R. et al., 2016; Xie et al., 2018). Studies have shown that non-dopaminergic neuron systems play an important role in the development of Parkinson’s disease (Guyenet and Crane, 1981; Bonnet, 2000; van der Heeden et al., 2014). METH exposure leads to the loss of neurons in the cerebral cortex and selectively damage pyramidal neurons in the CA3 region of the hippocampus and granule cells with dendritic cells activated by microglia (Kuczenski et al., 2007). In summary, these studies provide more potential targets for METH.

In the present study, we have demonstrated that CXCR1 expression is increased after METH exposure *in vivo* and *in vitro*. We also report that silence of CXCR1 expression using siRNA sequences can protect neuronal apoptosis caused by METH *in vitro* and promote the protective effect of astrocytes. We hypothesized that injury and protection coexist in the effect of astrocytes on METH-induced neuronal apoptosis. IL-8—CXCR1 may play an important role in this dynamic balance.

CXCR1 is a member of the G protein-coupled receptor family that is also a receptor for IL-8. CXCR1 binds IL-8 with high affinity and transduces the signal via the secondary messenger system activated by the G protein (Park et al., 2012; Risnik et al., 2017). Studies have shown that stimulating the neutrophil surface of CXCR1 can lead to neutrophil chemotaxis and activation (Ocana et al., 2017; Tavares et al., 2017), and inhibition of CXCR1 and CXCR2 can reduce the migration of neutrophils to tumor regions (Tavares et al., 2017). Furthermore, oncology research has suggested that blocking CXCR1 can inhibit the differentiation of breast cancer stem cells (Jia et al., 2017), and that CXCR1 is overexpressed in the development of malignant melanoma and involved in cell growth and angiogenesis (Uen et al., 2015; Jacquelot et al., 2016). In the meantime, other study has demonstrated that CX3CR1 can mediate neuronal apoptosis in a cerebral ischemic model, thus potentially playing a role in the pathophysiology of stroke (Wang et al., 2018).

In the current study, we found that the METH exposure increased the expression of CXCR1 *in vivo* and *in vitro*. Immunofluorescence co-localization experiments showed that CXCR1 was mainly expressed on neurons in the midbrain of METH-exposed mice. Besides, the induction of CXCR1 expression by METH occurred in a dose-dependent manner. Inhibition of CXCR1 expression by siRNA sequences also attenuated METH-induced neuronal apoptosis. To verify the role of CXCR1 in METH-induced neuronal apoptosis, we examined two important molecules involved in mitochondrial apoptosis, Bax and Bcl-2. Bax, a water-soluble protein homologous to Bcl-2, is an apoptosis-promoting gene in the Bcl-2 gene family. Bax overexpression can antagonize the protective effect of Bcl-2 and cause cell death (Kuwana et al., 2005; Chen C. et al., 2016). In our previous study, we found that METH exposure can result in the overexpression of Bax and the silencing of Bcl-2 through PUMA, which leads to the mitochondrial apoptotic pathway by releasing cytochrome (Chen C. et al., 2016). The present results showed that, after CXCR1 was inhibited, the expression of Bax was downregulated and the expression of Bcl-2 was upregulated. Meanwhile, the expression of cleaved caspase-3 and cleaved PARP were downregulated, indicating that CXCR1 can mediate METH-induced neuronal apoptosis through the mitochondrial apoptosis pathway.

IL-8 is also known as neutrophil chemokine. It induces target cell chemotaxis, primarily in neutrophils and other granulocytes, causing them to migrate toward the site of infection. IL-8 also induces phagocytosis and is considered to be a potent promoter of angiogenesis (Han et al., 2018; Paulitti et al., 2018). IL-8 can be secreted by any cell that has a Toll-like receptor that is associated with an innate immune response. In our previous study, it was found that Toll-like receptor four mediates METH-induced activation of astrocytes through the NF-κB and caspase-11 signaling pathways (Du et al., 2017). Thus, we detected the expression of IL-8 in U87MG cells and in the culture medium. Results showed that METH application caused the production and secretion of IL-8 in U87MG cells. Furthermore, we also demonstrated that METH exposure induced activation of the NF-κB pathway in U87MG cells, which regulated the expression of IL-8. These findings are consistent with previous studies that suggested that the NF-κB pathway was activated in primary cultured astrocytes exposed to 2.0 mM of METH.

Previous studies have focused on the role of METH in the context of one type of cell, through PUMA, IGFBP5 and other ways to induce neuronal apoptosis (Qiao et al., 2014; Chen C. et al., 2016); METH through NF-κB, caspase-11 and other ways to the induce activation of astrocytes was also evaluated (Du et al., 2017). However, under the stimulation of METH, one question that has not been answered is regarding what happens to neurons when astrocytes and neurons are co-cultured. Studies have found that glial cells involved in the dopaminergic neurons damage (Krencik et al., 2017; Ugbode et al., 2017; Facci et al., 2018). Astrocytes may affect neuronal conditions through diverse molecules such as a-syn or glutamate, with the effect possibly being either protection or damage (Gustafsson et al., 2017; Lee et al., 2017; Madji Hounoum et al., 2017; Yu et al., 2018). In the present study, we used a noncontact, sandwich-type neuron-astrocyte co-culture to study the effects of astrocytes on neurons under the action of METH. Results showed that the neuronal apoptosis was significant reduced in the co-culture group with 2.0 mM of METH for 24 h. This effect is similar to the research of PD, astrocytes rapidly engulf α-syn oligomers resulting in impaired mitochondria, thereby protecting neurons (Gustafsson et al., 2017). According to current research and previous studies (Du et al., 2017), METH treatment of astrocytes alone can cause activation of the TLR4—NF-kB pathway and increased expression of IL 1, 8 and 18. But according to the results of co-culture, astrocytes in the co-culture system protected the damaged neurons. Therefore, we speculate that astrocytes present different states under different processing conditions, and their neuroprotective and injury effects are dynamically balanced. In the present study, the overall performance of astrocytes is the protection state. However, the specific mechanism needs further research.

Other novel findings are that knockdown CXCR1 expression can reduce neuronal apoptosis in an increased fashion. To test whether IL-8 plays a role in this process, we administered exogenous recombinant IL-8 to SH-SY5Y cells in combination with METH. As a result, IL-8 was found to enhance METH-induced apoptosis and to significantly up-regulate CXCR1 expression. These data demonstrated that CXCR1 plays an important role in METH-induced neuronal apoptosis and that the IL-8 secreted by astrocytes is involved in activating CXCR1. The role of astrocytes in neuronal repair may be a double-sided and dynamically balanced process, with damage and protection existing simultaneously. Further studies are needed to illustrate the role of astrocytes.

## Conclusion

In summary, this study demonstrated that CXCR1 plays an important role in neuronal apoptosis induced by METH. The blockade of CXCR1 expression significantly attenuated METH-induced neuronal apoptosis and enhanced the protective effect of astrocytes on neurons. Moreover, we provide evidence that IL-8 plays a role in neuronal injury. The underlying mechanisms about how astrocytes play a neuroprotective role in the fact of METH exposure and whether other effects could occur requires further research.

## Data Availability

The authors declare that the data supporting the findings of this study are available within the article.

## Author Contributions

S-HD and WZ conducted all the experiments with the help of X-QL and X-HT. D-FQ and HW designed the research. S-HD, CL and XY analyzed and interpreted the results. D-FQ and S-HD wrote the manuscript.

## Conflict of Interest Statement

The authors declare that the research was conducted in the absence of any commercial or financial relationships that could be construed as a potential conflict of interest.

## Abbreviations

Bay 11–7082: ((E)3-[(4-methylphenyl)-sulfonyl]-2-propenenitrile
CNS: central nervous system
CXCR1: C-X-C motif chemokine receptor 1
DMEM: Dulbecco’s modified Eagle’s medium
FBS: Fetal bovine serum
IKK: IκB kinase
IL: Interleukin
METH: Methamphetamine
NF-κB: nuclear factor-kappa B
PARP: poly(ADP-ribose) polymerase
VDF: Polyvinylidenedifluoride
SDS-PAGE: Sodium dodecyl sulfate polyacrylamide gel.
